# Prediction of swallowing improvement after tracheal reconstruction in patients receiving LFES-containing rehabilitation: a retrospective cohort study

**DOI:** 10.3389/fsurg.2026.1909631

**Published:** 2026-10-07

**Authors:** Yunjuan Liang, Jinfen Hu, Yuying Liang, Xiaoying Xia

**Affiliations:** Department of Thoracic Surgery, The First Affiliated Hospital of Guangzhou Medical University, Guangzhou, Guangdong, China

**Keywords:** dysphagia, low-frequency electrical stimulation, prediction model, prognosis, tracheal reconstruction

## Abstract

**Background:**

Dysphagia is a clinically important complication after tracheal reconstruction. Low-frequency electrical stimulation (LFES) supports postoperative swallowing rehabilitation, yet swallowing outcomes vary substantially among patients. We aimed to identify prognostic factors associated with observed swallowing improvement and to develop an internally validated clinical prediction model among patients receiving LFES-containing rehabilitation after tracheal reconstruction.

**Methods:**

This retrospective cohort study included 200 patients with postoperative dysphagia who received LFES-containing rehabilitation. Patients were grouped by prespecified criteria for swallowing improvement after rehabilitation. Demographic, perioperative, swallowing-related, and prespecified treatment-plan variables available before rehabilitation were evaluated. The cohort was divided by outcome-stratified random sampling into training and independent validation sets. Candidate predictors were examined using univariable logistic regression, LASSO, random forest, and XGBoost, followed by multivariable logistic regression. Model performance was assessed using ROC analysis, calibration, bootstrap internal validation, and exploratory decision-curve, subgroup, and sensitivity analyses.

**Results:**

Among 200 patients, 126 (63.0%) met the prespecified swallowing-improvement criteria. Older age (OR = 0.96, 95% CI: 0.93–0.98) and aspiration (OR = 0.41, 95% CI: 0.18–0.88) were independently associated with a lower probability of improvement, whereas a greater planned number of LFES sessions was associated with a higher probability of improvement (OR = 1.09, 95% CI: 1.04–1.16). The logistic regression model achieved AUCs of 0.776 in the training set and 0.767 in the independent validation set, with acceptable calibration. The optimism-corrected AUC after 500 bootstrap resamples was 0.721. Exploratory decision-curve analysis suggested potential net benefit across portions of the evaluated threshold-probability range. XGBoost achieved the numerically highest validation AUC (0.780), followed by logistic regression (0.765) and random forest (0.760), with only small absolute differences among these models. Main associations were generally consistent across sensitivity analyses.

**Conclusion:**

Among patients receiving LFES-containing rehabilitation after tracheal reconstruction, older age and aspiration were associated with a lower probability of observed swallowing improvement, whereas a greater planned number of LFES sessions was associated with a higher probability of improvement. The model demonstrated moderate discrimination and may provide a preliminary framework for prognostic risk stratification and identifying patients who warrant closer rehabilitation monitoring. External validation is required before routine clinical use.

## 1 introduction

Tracheal reconstruction is primarily performed to manage tracheal or laryngotracheal stenosis caused by benign or malignant airway lesions (1, 2). Large cohort studies have reported that the main indications for tracheal and laryngotracheal resection and reconstruction include post-intubation tracheal stenosis, tracheal tumors, idiopathic laryngotracheal stenosis, and tracheoesophageal fistula, accounting for approximately 65.4%, 23.1%, 9.2%, and 2.3% of cases, respectively (3). With advances in perioperative management, anesthetic techniques, and intensive care, the safety and success rates of tracheal reconstruction have steadily improved (4–6). Nevertheless, because the procedure involves airway reconstruction, laryngotracheal manipulation, adjacent neurovascular structures, and postoperative airway management, patients remain at risk of various complications. Complications following tracheal reconstruction include anastomotic dehiscence, benign stenosis or restenosis, tracheoesophageal fistula, tracheocutaneous fistula, laryngeal edema, recurrent laryngeal nerve paralysis, and dysphagia (7).

Dysphagia is an important complication following tracheal reconstruction, although its reported incidence varies considerably across studies (8). In patients undergoing long-segment tracheal resection and reconstruction, postoperative swallowing and speech dysfunction has been reported in 32.2% of cases, increasing to 68.0% among those requiring laryngeal release (9). In another study of subglottic stenosis, swallowing impairment occurred one week postoperatively in approximately 80.4% of patients undergoing mini infrahyoid release and in 100% of those undergoing full release; notably, 80% of patients in the full-release group continued to experience choking symptoms one month after surgery (10). Although postoperative dysphagia is often transient, recovery may be prolonged in older patients and in those undergoing laryngeal release, stent placement, or complex airway reconstruction. Potential contributing factors include surgical manipulation of the airway, nerve injury, prolonged intubation, and intensive care unit admission. Therefore, effective swallowing rehabilitation is essential for improving functional recovery and quality of life in this population.

Low-frequency electrical stimulation (LFES), a form of neuromuscular electrical stimulation, has been used for conditions such as low back pain, chronic musculoskeletal pain, post-stroke hemiplegia, and cancer-related symptoms (11–14). In recent years, LFES has also been incorporated into rehabilitation strategies for swallowing dysfunction. By stimulating the suprahyoid and pharyngeal muscles and related neuromuscular pathways, LFES may facilitate muscle activation and swallowing coordination and may enhance swallowing-related neuromuscular responsiveness (15). However, evidence regarding its use in patients with dysphagia after tracheal reconstruction remains limited, and swallowing outcomes vary substantially among patients receiving LFES-containing rehabilitation. Clinical characteristics such as age, comorbidities, nutritional status, postoperative pulmonary infection, baseline swallowing function, timing of rehabilitation initiation, and prespecified treatment-plan characteristics may be associated with the likelihood of observed swallowing improvement. At present, evidence is limited regarding clinically interpretable tools for estimating the probability of swallowing improvement in this specific population. A prediction model based on routinely available clinical variables may therefore provide a framework for preliminary prognostic risk stratification and help identify patients who may require closer clinical monitoring and reassessment during rehabilitation.

We therefore conducted a retrospective cohort study of patients who developed dysphagia after tracheal reconstruction and subsequently received LFES-containing rehabilitation. We evaluated demographic characteristics, comorbidities, perioperative factors, baseline swallowing assessments, and prespecified treatment-plan variables associated with observed swallowing improvement and developed an internally validated clinical prediction model. The model was designed to estimate the probability of observed swallowing improvement within an LFES-treated cohort and to support preliminary prognostic stratification. It was not intended to estimate the causal effect of LFES, determine treatment eligibility, or prescribe rehabilitation intensity, and external validation is required before consideration for routine clinical use.

## 2 Methods

### 2.1 Study design and participants

This retrospective cohort study included patients who underwent tracheal reconstruction at our center between January 2022 and May 2026 and subsequently developed postoperative dysphagia. During the study period, 278 potentially eligible patients were identified and assessed according to the prespecified inclusion and exclusion criteria. We excluded 18 patients with pre-existing dysphagia, 14 patients with severe neurological disorders that substantially affected swallowing function, 12 patients who were unable to complete the planned postoperative rehabilitation program because of severe complications, 21 patients because of substantial missing clinical or swallowing-assessment data, and 13 patients because of incomplete follow-up. The final analytical cohort therefore comprised 200 patients who received LFES-containing rehabilitation. According to the prespecified post-rehabilitation outcome criteria, patients were classified as having swallowing improvement or no swallowing improvement.

### 2.2 Inclusion and exclusion criteria

The inclusion criteria were: (1) tracheal reconstruction; (2) postoperative dysphagia confirmed by swallowing-function assessment; (3) receipt of LFES-containing postoperative rehabilitation; and (4) availability of the clinical, perioperative, and pre- and post-rehabilitation swallowing assessments required to determine the study outcome. Exclusion criteria were pre-existing dysphagia; severe stroke, Parkinson's disease, myasthenia gravis, or other neurological disorders likely to substantially affect swallowing function; inability to complete the planned postoperative rehabilitation program because of severe complications; substantial missing clinical or swallowing-assessment data; or incomplete follow-up.

Missingness was assessed for each candidate predictor before model development. No missing values were present among the candidate predictors in the final 200-patient analytical cohort; therefore, no statistical imputation was required. Patients excluded because of substantial missing information were removed before the training/validation split. Predictor-level missingness is summarized in the Supplementary File.

### 2.3 LFES-containing rehabilitation

All enrolled patients received LFES within a postoperative swallowing rehabilitation program. LFES was delivered using a surface neuromuscular electrical stimulation device at 80 Hz with a biphasic pulsed-current waveform and a pulse width of 700 microseconds. Stimulation intensity was individually adjusted to produce perceptible stimulation and/or mild local muscle contraction without pain. Electrode placement was individualized according to the type and severity of swallowing dysfunction and could include the anterior cervical region, suprahyoid muscles, or surface projection areas of relevant pharyngeal muscles. Sessions lasted approximately 20–30 min and were generally delivered five times weekly. The planned rehabilitation course lasted 2–4 weeks and was individualized according to the rehabilitation protocol and the patient's clinical condition. Planned and completed session numbers were recorded. In addition to LFES, patients received routine swallowing-related care and safety education as clinically indicated. Some patients received additional structured swallowing training, including oral-motor exercises, tongue and pharyngeal muscle exercises, swallowing-reflex facilitation, feeding-posture guidance, and safe-swallowing training; this intervention was recorded separately. LFES was delivered by qualified rehabilitation physicians. Adherence was defined as completion of at least 80% of the planned sessions. Treatment interruptions, reasons for discontinuation, and LFES-related adverse events were documented when applicable. Treatment-related information included the interval from surgery to LFES initiation, planned and completed LFES sessions, treatment duration, adherence, and additional structured swallowing training. Only variables available before rehabilitation initiation or as part of the prespecified treatment plan were considered candidate prognostic predictors. The planned number of LFES sessions was therefore treated as a pre-rehabilitation treatment-plan variable and not as a measure of received treatment dose or as an estimate of the causal effect of LFES.

### 2.4 Clinical data collection

Patient data were extracted from electronic medical and rehabilitation records. Demographic and clinical variables included age, sex, body mass index (BMI), hypertension, diabetes mellitus, chronic obstructive pulmonary disease, and serum albumin level. Perioperative and airway-related variables included preoperative tracheotomy status, postoperative endotracheal-intubation duration, tracheal-stenosis length, operation time, mechanical-ventilation duration, intensive care unit (ICU) stay, and postoperative pulmonary infection. Swallowing-related variables included Functional Oral Intake Scale (FOIS) and Standardized Swallowing Assessment (SSA) scores obtained after surgery and immediately before rehabilitation, as well as aspiration status. Post-rehabilitation FOIS and SSA assessments were used to determine the outcome. Resumption of safe oral feeding was recorded as a descriptive post-rehabilitation outcome and was not used as a candidate predictor. Treatment-plan variables included time from surgery to LFES initiation, planned LFES sessions, and additional structured swallowing training.

### 2.5 Outcome definition

The primary outcome was observed swallowing improvement after LFES-containing rehabilitation. At completion of the planned rehabilitation course, pretreatment and post-rehabilitation swallowing assessments were compared. Improvement was defined as meeting at least one of the following prespecified criteria: (1) an increase of ≥2 levels in FOIS; (2) a reduction of ≥9 points in SSA; or (3) documented improvement in aspiration or choking, defined as a clear reduction or disappearance of previously documented aspiration or choking at post-rehabilitation assessment. Patients meeting at least one criterion were classified as having swallowing improvement, whereas all others were classified as having no swallowing improvement. Resumption of oral feeding was recorded descriptively and was not an independent component of the primary outcome.

The FOIS- and SSA-defined components were directly reconstructible from the retained pretreatment and post-rehabilitation scores. The aspiration/choking component had originally been assigned during chart abstraction from contemporaneous medical, rehabilitation, and swallowing-assessment documentation; however, a separate structured post-rehabilitation aspiration/choking variable was not retained in the analytical dataset. Therefore, this component could not be independently reconstructed from the retained analysis file alone. Two patients met the composite improvement outcome without meeting either the FOIS or SSA threshold. Jinfen Hu re-reviewed the original source records for these two patients, and the original classification based on documented improvement in aspiration/choking was confirmed in both cases.

### 2.6 Statistical analysis

All statistical analyses were performed using R software version 4.4.3. Continuous variables were assessed for normality before analysis. Normally distributed variables are presented as mean ± standard deviation and were compared using the independent-samples t test, whereas non-normally distributed variables are presented as median and interquartile range and were compared using the Mann–Whitney *U*-test. Categorical variables are presented as frequencies and percentages and were compared using the chi-square test or Fisher's exact test, as appropriate. All statistical tests were two-sided, and *P* < 0.05 was considered statistically significant. Before model development, the 200-patient analytical cohort was randomly divided into a training set (70%) and an independent validation set (30%) using outcome-stratified sampling with a fixed random seed of 2026. All predictor screening, variable selection, model fitting, and model tuning were performed using the training set only. The validation set was not used for predictor selection, coefficient estimation, preprocessing-parameter estimation, or hyperparameter tuning. Univariable logistic regression was first performed in the training set to examine associations between candidate predictors and observed swallowing improvement. Candidate predictors were additionally evaluated using least absolute shrinkage and selection operator (LASSO) regression, random forest variable importance, and XGBoost variable importance. LASSO tuning was performed by cross-validation within the training set. Random forest variable importance was derived exclusively from the training data, and XGBoost tuning, including selection of the number of boosting iterations, was performed using five-fold cross-validation within the training set. Predictor selection integrated the training-set results from these approaches with prespecified clinical relevance. The final predictor set was locked before evaluation of validation-set performance and was entered into a multivariable logistic regression model fitted using the training set. Multicollinearity was assessed using variance inflation factors. The fitted logistic regression model was subsequently applied without modification to the independent validation set. Discrimination was assessed using receiver operating characteristic (ROC) curves and the area under the curve (AUC) with 95% confidence intervals. The classification threshold was selected once in the training set using the Youden index and was then applied unchanged to the validation set; no threshold optimization was performed in the validation data. Sensitivity, specificity, accuracy, positive predictive value, and negative predictive value were calculated at this locked threshold.

### 2.7 Machine learning model comparison and sensitivity analysis

For comparison with the primary logistic regression model, K-nearest neighbors (KNN), random forest, support vector machine (SVM), and XGBoost models were also evaluated. A separate logistic regression comparator was also fitted using the broader candidate-predictor set, whereas the primary logistic regression model included the eight selected predictors. All model-specific preprocessing and hyperparameter tuning were performed exclusively within the training set using internal cross-validation. After model development and tuning were completed, each model was applied without further modification to the independent validation set. Validation-set area under the receiver operating characteristic curve (AUC) values were used to describe comparative discrimination. Because these comparisons were exploratory and the sample size was limited, numerical differences in AUC were interpreted descriptively rather than as evidence of statistically superior performance.

For the primary logistic regression model, calibration was evaluated using calibration plots and the Hosmer–Lemeshow goodness-of-fit test. Bootstrap internal validation with 500 resamples was performed within the training set to estimate optimism-corrected discrimination. Decision curve analysis was conducted as an exploratory assessment of potential net benefit across a range of threshold probabilities. Because no external validation or prospective impact evaluation was performed, the decision-curve findings were interpreted as evidence of potential rather than established clinical utility.

Exploratory subgroup analyses were conducted to examine the associations of selected prognostic factors with observed swallowing improvement across clinically relevant strata defined by age, sex, postoperative pulmonary infection, aspiration status, and receipt of additional structured swallowing training. These analyses were conducted separately from prediction-model development and were not used for predictor selection, model tuning, or validation. Differences in statistical significance between strata were not considered evidence of effect modification in the absence of formal interaction testing.

Sensitivity analyses were conducted after excluding patients aged ≥80 years, excluding patients with postoperative pulmonary infection, and restricting the cohort to patients with a BMI of 18.5–32 kg/m^2^. The direction and magnitude of the resulting associations were compared with those observed in the primary analysis. Because the planned number of LFES sessions was determined by the treating clinician before rehabilitation and could reflect clinical treatment-planning decisions or confounding by indication, an additional sensitivity analysis was performed by refitting the multivariable model after excluding planned LFES sessions. These analyses were intended to assess the robustness of the prognostic associations and were not interpreted as estimates of LFES treatment effects.

To assess the reproducibility and robustness of the composite primary outcome, exploratory component-specific sensitivity analyses were conducted using the FOIS- and SSA-defined components that could be independently reconstructed from the retained analytical dataset. FOIS-defined improvement was defined as an increase of ≥2 levels from pretreatment to post-rehabilitation assessment, whereas SSA-defined improvement was defined as a reduction of ≥9 points. The frequency and overlap of these components were summarized, and separate multivariable logistic regression models were fitted using the same prespecified covariate set as in the primary analysis. These component-specific analyses were not used to redefine the primary outcome and did not contribute to predictor selection, model tuning, or modification of the primary prediction model.

Because a separate structured post-rehabilitation aspiration/choking variable was not retained in the analytical dataset, this component could not be independently reconstructed for component-specific regression analysis from the retained analysis file alone. The two composite responders who met neither the FOIS nor SSA threshold were therefore re-reviewed against the original source records by Jinfen Hu, and the original classifications based on documented improvement in aspiration/choking were confirmed in both cases.

## 3 Results

### 3.1 Baseline characteristics of the participants

Among the 200 patients who underwent tracheal reconstruction and received LFES-containing rehabilitation, 126 (63.0%) met the predefined criteria for swallowing improvement, whereas 74 (37.0%) did not. Patients in the swallowing improvement group were significantly younger than those in the no swallowing improvement group (48.62 ± 14.26 vs. 58.59 ± 16.60 years, *P* < 0.001). No significant between-group differences were observed in sex, body mass index, hypertension, diabetes mellitus, chronic obstructive pulmonary disease, preoperative tracheotomy, intubation time, stenosis length, operation time, duration of mechanical ventilation, or intensive care unit stay. Aspiration was less frequent in the swallowing improvement group than in the no swallowing improvement group (43.7% vs. 62.2%, *P* = 0.017). The pretreatment SSA score was also lower in the swallowing improvement group (38.63 ± 10.05 vs. 43.38 ± 10.97, *P* = 0.002), indicating less severe swallowing impairment before rehabilitation. In addition, patients with swallowing improvement had a greater planned number of LFES sessions (18.56 ± 7.47 vs. 13.93 ± 7.66, *P* < 0.001). No significant difference was observed in the receipt of additional structured swallowing training between the two groups. Resumption of oral feeding was observed in 83.3% of patients in the swallowing improvement group compared with 27.0% of those in the no swallowing improvement group (*P* < 0.001). Because resumption of oral feeding was analyzed as an additional descriptive swallowing outcome rather than as an independent predictor, this finding was interpreted descriptively. Overall, younger age, absence of aspiration, lower pretreatment SSA scores, and a greater planned number of LFES sessions were associated with observed swallowing improvement among patients receiving LFES-containing rehabilitation (Table 1).

**Table 1:** Baseline characteristics of patients receiving LFES-containing rehabilitation after tracheal reconstruction.

Characteristic	Overall (*N* = 200)	Improvement (*N* = 126)	Non-improvement (*N* = 74)	*P*_value
Age	52.31 (15.88)	48.62 (14.26)	58.59 (16.60)	<0.001
Sex				
Female	97 (48.5)	58 (46.0)	39 (52.7)	0.444
Male	103 (51.5)	68 (54.0)	35 (47.3)	
BMI	24.48 (3.73)	24.33 (3.72)	24.74 (3.77)	0.454
Hypertension				
No	85 (42.5)	59 (46.8)	26 (35.1)	0.143
Yes	115 (57.5)	67 (53.2)	48 (64.9)	
Diabetes				
No	98 (49.0)	61 (48.4)	37 (50.0)	0.944
Yes	102 (51.0)	65 (51.6)	37 (50.0)	
COPD				
No	110 (55.0)	66 (52.4)	44 (59.5)	0.410
Yes	90 (45.0)	60 (47.6)	30 (40.5)	
Preoperative tracheotomy				
No	97 (48.5)	61 (48.4)	36 (48.6)	1.000
Yes	103 (51.5)	65 (51.6)	38 (51.4)	
Intubation time (d)	9.18 (5.71)	9.06 (5.76)	9.38 (5.68)	0.708
Stenosis length (cm)	3.42 (1.26)	3.38 (1.26)	3.47 (1.27)	0.619
Operation time (min)	271.42 (90.82)	268.81 (88.83)	275.86 (94.55)	0.597
Mechanical ventilation time (h)	69.26 (29.02)	67.60 (30.52)	72.08 (26.23)	0.293
ICU time (d) Mean ± SD	7.00 (4.17)	7.12 (4.26)	6.80 (4.03)	0.599
Initial FOIS Score After Surgery Mean ± SD	2.40 (1.19)	2.29 (1.24)	2.58 (1.10)	0.101
Initial SSA Score After Surgery Mean ± SD	42.80 (10.76)	42.79 (11.07)	42.82 (10.27)	0.981
Aspiration				
No	99 (49.5)	71 (56.3)	28 (37.8)	0.017
Yes	101 (50.5)	55 (43.7)	46 (62.2)	
Albumin (g/L) Mean ± SD	36.81 (4.80)	36.66 (4.73)	37.07 (4.93)	0.557
Lung infection				
No	105 (52.5)	67 (53.2)	38 (51.4)	0.918
Yes	95 (47.5)	59 (46.8)	36 (48.6)	
Time to Low Frequency Treatment Initiation days Mean ± SD	5.62 (2.83)	5.48 (2.84)	5.85 (2.81)	0.377
Planned_treatment_sessions Mean ± SD	16.85 (7.85)	18.56 (7.47)	13.93 (7.66)	<0.001
Combined swallowing training				
No	105 (52.5)	63 (50.0)	42 (56.8)	0.437
Yes	95 (47.5)	63 (50.0)	32 (43.2)	
Pretreatment FOIS Score Mean ± SD	2.58 (1.25)	2.54 (1.20)	2.66 (1.34)	0.506
Pretreatment SSA Score Mean ± SD	40.38 (10.62)	38.63 (10.05)	43.38 (10.97)	0.002
Resumption of oral feeding by end of rehabilitation				
No	75 (37.5)	21 (16.7)	54 (73.0)	<0.001
Yes	125 (62.5)	105 (83.3)	20 (27.0)	

BMI, body mass index; Mean ± SD: mean ± standard deviation; COPD:Chronic Obstructive Pulmonary Disease; ICU:Intensive Care Unit; FOIS:Functional Oral Intake Scale; SSA:Standardized Swallowing Assessment;d:day.

### 3.2 Univariate logistic regression analysis of factors associated with swallowing improvement

As shown in Table 2, univariable logistic regression analysis performed in the training set identified age (OR = 0.96, 95% CI: 0.94–0.98, *P* < 0.001), aspiration (OR = 0.39, 95% CI: 0.19–0.78, *P* = 0.008), and the planned number of LFES sessions (OR = 1.08, 95% CI: 1.03–1.13, *P* = 0.002) as factors significantly associated with observed swallowing improvement. Older age and the presence of aspiration were associated with a lower probability of swallowing improvement, whereas a greater planned number of LFES sessions was associated with a higher probability of improvement. Pretreatment SSA score showed an inverse association that did not reach statistical significance (OR = 0.97, 95% CI: 0.94–1.00, *P* = 0.059). Sex, BMI, hypertension, diabetes mellitus, chronic obstructive pulmonary disease, preoperative tracheotomy, intubation duration, stenosis length, operation time, mechanical ventilation duration, ICU stay, initial postoperative FOIS and SSA scores, serum albumin level, postoperative pulmonary infection, time to LFES initiation, additional structured swallowing training, and pretreatment FOIS score were also not significantly associated with swallowing improvement in the univariable analyses (all *P* > 0.05).

**Table 2:** Univariable logistic regression of factors associated with observed swallowing improvement.

Variable	OR (95%CL)	P_value
Age	0.96 (0.94–0.98)	<0.001
BMI	1.00 (0.91,1.09)	0.956
Hypertension	0.70 (0.35,1.40)	0.314
Diabetes	0.99 (0.50,1.97)	0.978
COPD	1.58 (0.79,3.18)	0.196
Preoperative tracheotomy	1.00 (0.50,1.99)	0.992
Intubation time (d)	0.97 (0.91,1.03)	0.319
Stenosis length (cm)	0.90 (0.68,1.18)	0.443
Operation time (min)	1.00 (0.99,1.00)	0.568
Mechanical ventilation time (h)	1.00 (0.99,1.01)	0.636
ICU time (d)	0.99 (0.91,1.07)	0.738
Initial FOIS score after surgery	0.92 (0.68,1.23)	0.563
Initial SSA score after surgery	1.00 (0.97,1.03)	0.958
Aspiration	0.39 (0.19,0.77)	0.008
Albumin (g/L)	1.00 (0.93,1.07)	0.948
Lung infection	1.21 (0.60,2.43)	0.598
Time to low-frequency treatment initiation (d)	0.95 (0.85,1.07)	0.409
Planned treatment sessions	1.08 (1.03,1.13)	0.002
Combined swallowing training	1.14 (0.57,2.27)	0.707
Pretreatment FOIS score	0.92 (0.70,1.20)	0.545
Pretreatment SSA score	0.97(0.94,1.00)	0.059

BMI, body mass index; Mean ± SD: mean ± standard deviation; COPD, Chronic Obstructive Pulmonary Disease; ICU, Intensive Care Unit; FOIS, Functional Oral Intake Scale; SSA, Standardized Swallowing Assessment; d:day.

### 3.3 Multivariable logistic regression analysis of factors associated with swallowing improvement

To identify independent factors associated with swallowing improvement, candidate predictors were evaluated using LASSO regression, random forest, and XGBoost. The findings from these approaches were integrated with univariable regression and clinical relevance. Eight variables were selected for the multivariable model: age, initial postoperative SSA score, aspiration, serum albumin level, postoperative pulmonary infection, time to LFES initiation, planned LFES sessions, and additional structured swallowing training (Figure 1).

**Figure 1:**
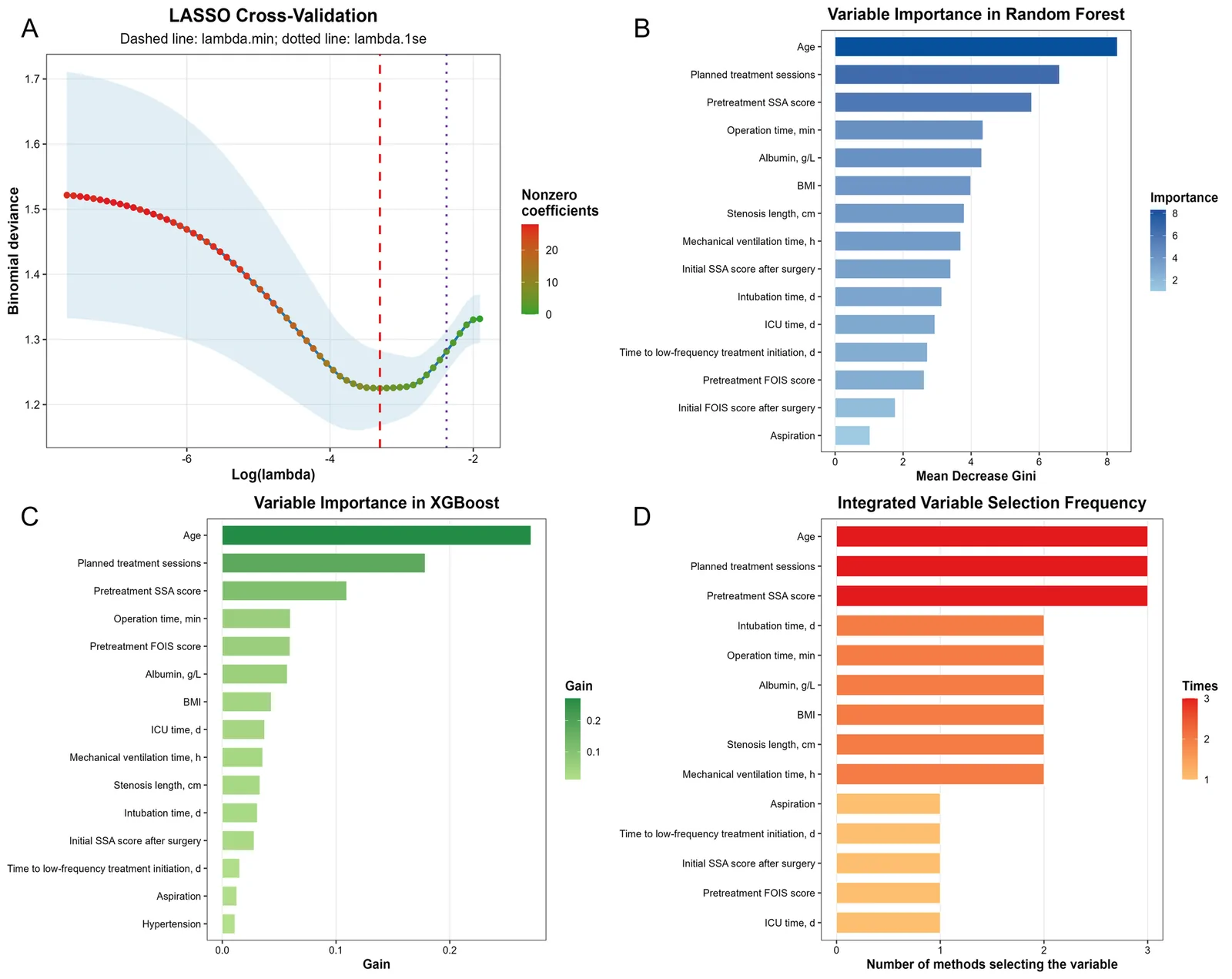
Screening of predictors of observed swallowing improvement. **(A)** LASSO regression screening variables. **(B)** Random forest model for selecting variables. **(C)** XGBoost model for selecting variables. **(D)** The variable selection results of combining LASSO regression, random forest and XGBoost methods.

The eight variables were entered into the multivariable logistic regression model. As shown in Table 3 and Figure 2, older age, aspiration, and planned LFES sessions were independently associated with observed swallowing improvement. Older age was associated with a lower probability of improvement (OR = 0.96, 95% CI: 0.93–0.98; *P* < 0.001), as was aspiration (OR = 0.41, 95% CI: 0.18–0.88; *P* = 0.023). A higher planned number of LFES sessions was associated with a higher probability of improvement (OR = 1.10, 95% CI: 1.04–1.16; *P* = 0.001). Initial postoperative SSA score, serum albumin level, postoperative pulmonary infection, time to LFES initiation, and additional structured swallowing training were not independently associated with improvement (all *P* > 0.05). All VIFs were below 2, indicating no substantial multicollinearity (Supplementary File).

**Table 3:** Multivariable logistic regression of factors associated with observed swallowing improvement.

Variable	OR (95%Cl)	P_value
Age	0.96 (0.93,0.98)	<0.001
Initial SSA score after surgery	0.99 (0.96,1.03)	0.792
Aspiration	0.41 (0.18,0.88)	0.023
Albumin (g/L)	1.02 (0.94,1.10)	0.688
Lung infection	1.32 (0.60,2.99)	0.492
Time to low-frequency treatment initiation (d)	0.89 (0.78,1.02)	0.108
Planned treatment sessions	1.10 (1.04,1.16)	0.001
Combined swallowing training	1.42 (0.63,3.23)	0.398

SSA, Standardized Swallowing Assessment;d:day.

**Figure 2:**
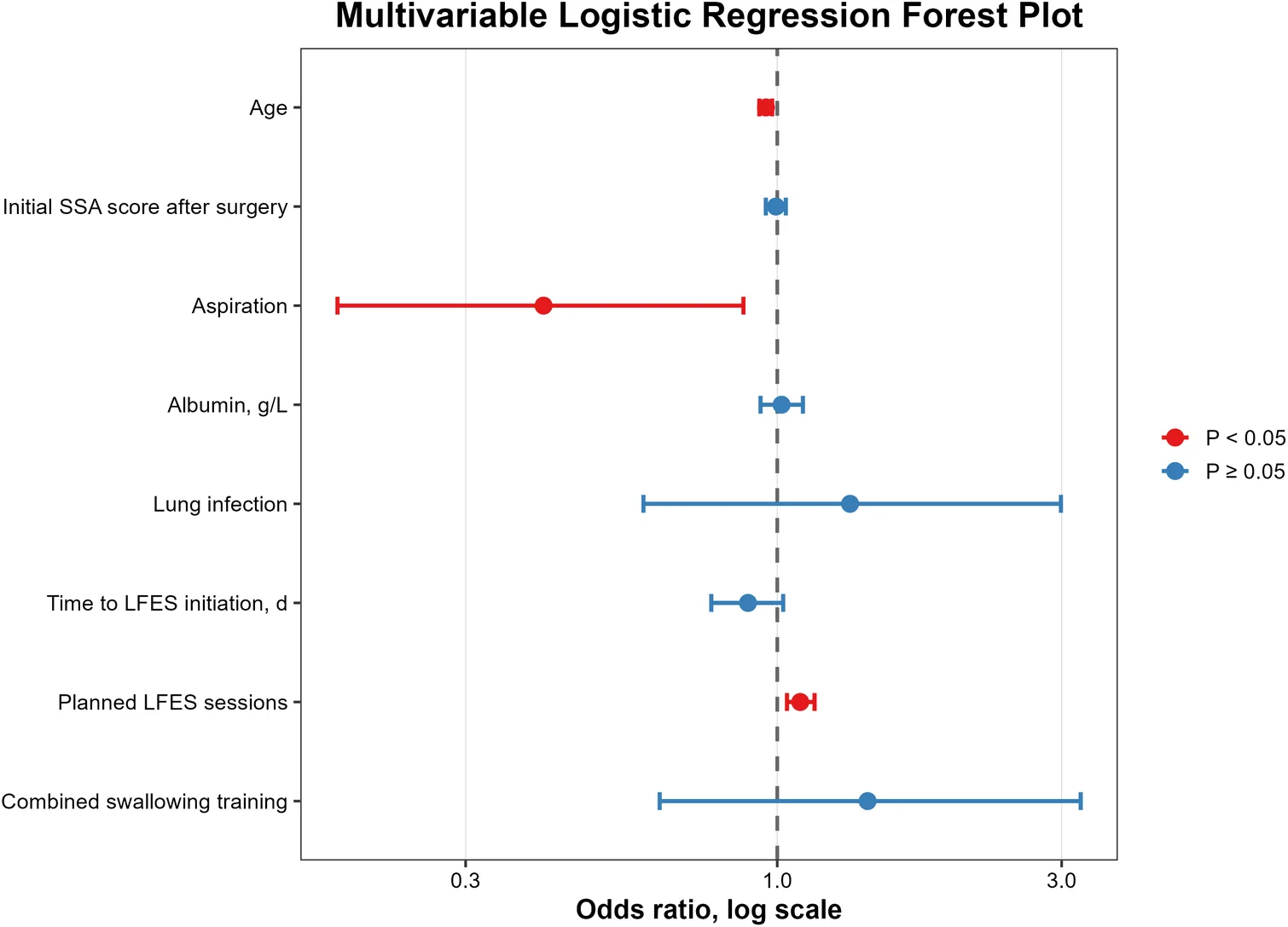
Forest plot of multivariate logistic regression analysis.

### 3.4 Model performance evaluation and visualization

ROC curve analysis showed that the logistic regression model achieved an AUC of 0.776 (95% CI: 0.70–0.85) in the training set and 0.767 (95% CI: 0.62–0.91) in the independent validation set, indicating moderate discriminative performance (Figure 3A). The optimal probability threshold was determined in the training set using the Youden index and was 0.679. At this threshold, the sensitivity, specificity, accuracy, positive predictive value, and negative predictive value were 0.663, 0.808, 0.716, 0.855, and 0.583, respectively, in the training set. The same threshold was subsequently applied unchanged to the validation set, yielding a sensitivity of 0.595, specificity of 0.773, accuracy of 0.661, positive predictive value of 0.815, and negative predictive value of 0.531. Calibration curves showed generally acceptable agreement between predicted and observed probabilities of swallowing improvement in both the training and validation sets (Figure 3B). The Hosmer–Lemeshow test showed no evidence of lack of fit in either the training set (*χ*^2^ = 5.768, df = 8, *P* = 0.673) or the validation set (*χ*^2^ = 11.589, df = 8, *P* = 0.170) (Supplementary File). Exploratory decision curve analysis suggested potential net benefit of the model relative to the treat-all and treat-none strategies across portions of the evaluated threshold-probability range (Figure 3C); however, these findings should not be interpreted as evidence of established clinical utility. The model coefficient plot illustrated the direction and magnitude of associations between the included predictors and the probability of observed swallowing improvement (Figure 3D). Positive coefficients were observed for additional structured swallowing training, planned LFES sessions, serum albumin level, and postoperative pulmonary infection, whereas negative coefficients were observed for initial postoperative SSA score, age, time to LFES initiation, and aspiration. These coefficient directions should be interpreted together with the corresponding confidence intervals and statistical significance reported in the multivariable regression analysis. Internal validation of the logistic regression model was additionally performed in the training set using 500 bootstrap resamples. The optimism-corrected AUC was 0.721, indicating moderate discrimination after correction for model optimism (Figure 3E). The reduction from the apparent to the optimism-corrected performance suggests some degree of model optimism and supports cautious interpretation pending external validation.

**Figure 3:**
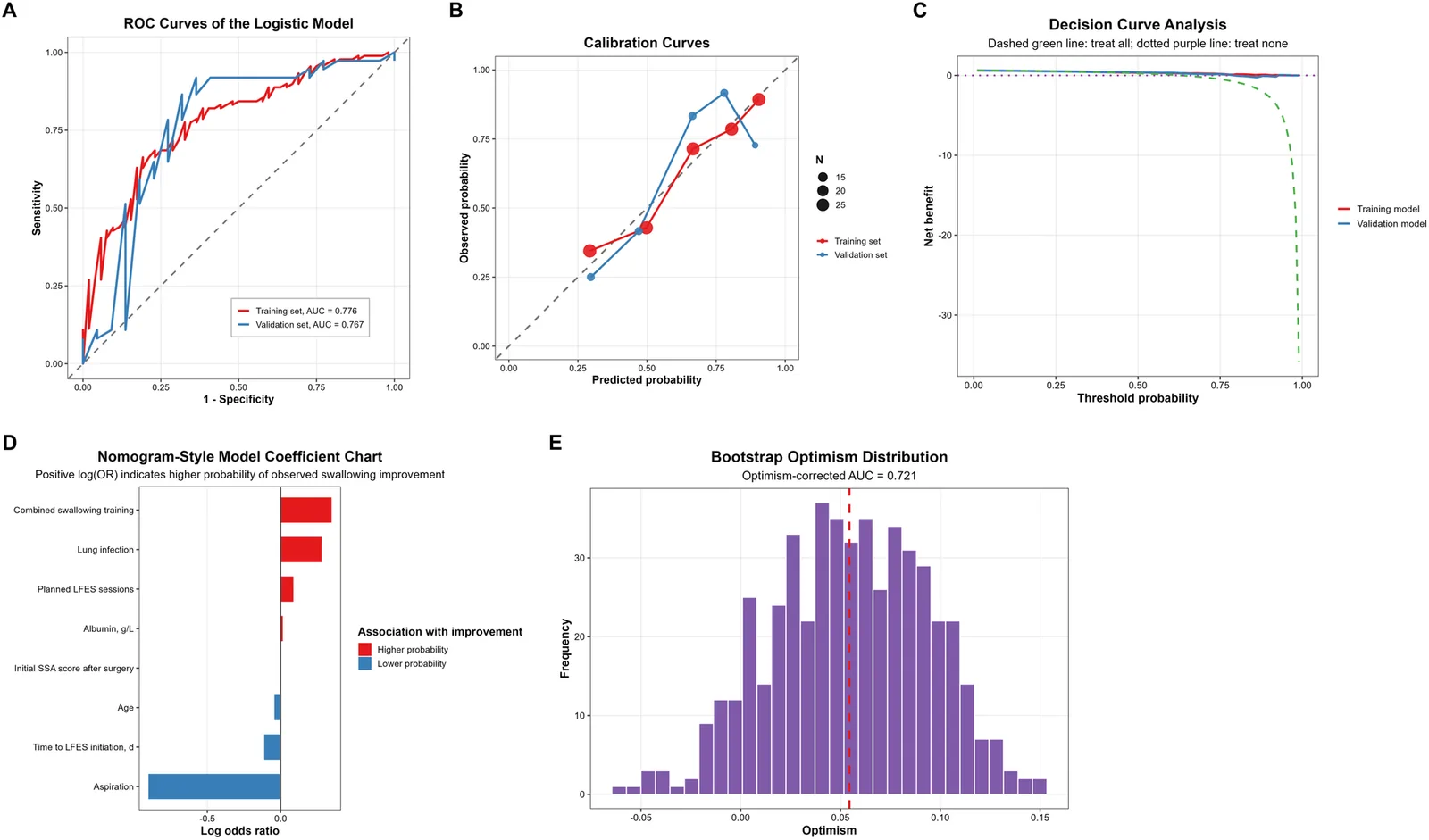
Performance evaluation of logistic regression prediction models. **(A)** ROC curves of the Logistic Regression model on the training and validation sets. **(B)** Calibration curves for the training and validation sets. **(C)** Decision curve analysis. **(D)** Logistic regression model coefficient diagram. **(E)** Bootstrap internal validation results.

### 3.5 Comparison of predictive performance among machine learning models

To compare predictive performance across different modeling approaches, logistic regression, K-nearest neighbors (KNN), random forest, support vector machine (SVM), and XGBoost models were developed and tuned using the training set and subsequently evaluated in the independent validation set. Receiver operating characteristic curves were generated for each model. XGBoost achieved the numerically highest validation AUC (0.780), followed by logistic regression (0.765), random forest (0.760), SVM (0.722), and KNN (0.636) (Figure 4). The logistic regression comparator used the broader candidate-predictor set and was fitted separately from the primary eight-predictor model; their validation AUCs were 0.765 and 0.767, respectively. Although XGBoost showed slightly higher discrimination than logistic regression, the absolute difference in AUC was small. Given the limited sample size and the greater interpretability of the logistic regression model, these findings suggest that the more complex machine-learning approaches provided limited incremental predictive value over conventional logistic regression in this cohort.

**Figure 4:**
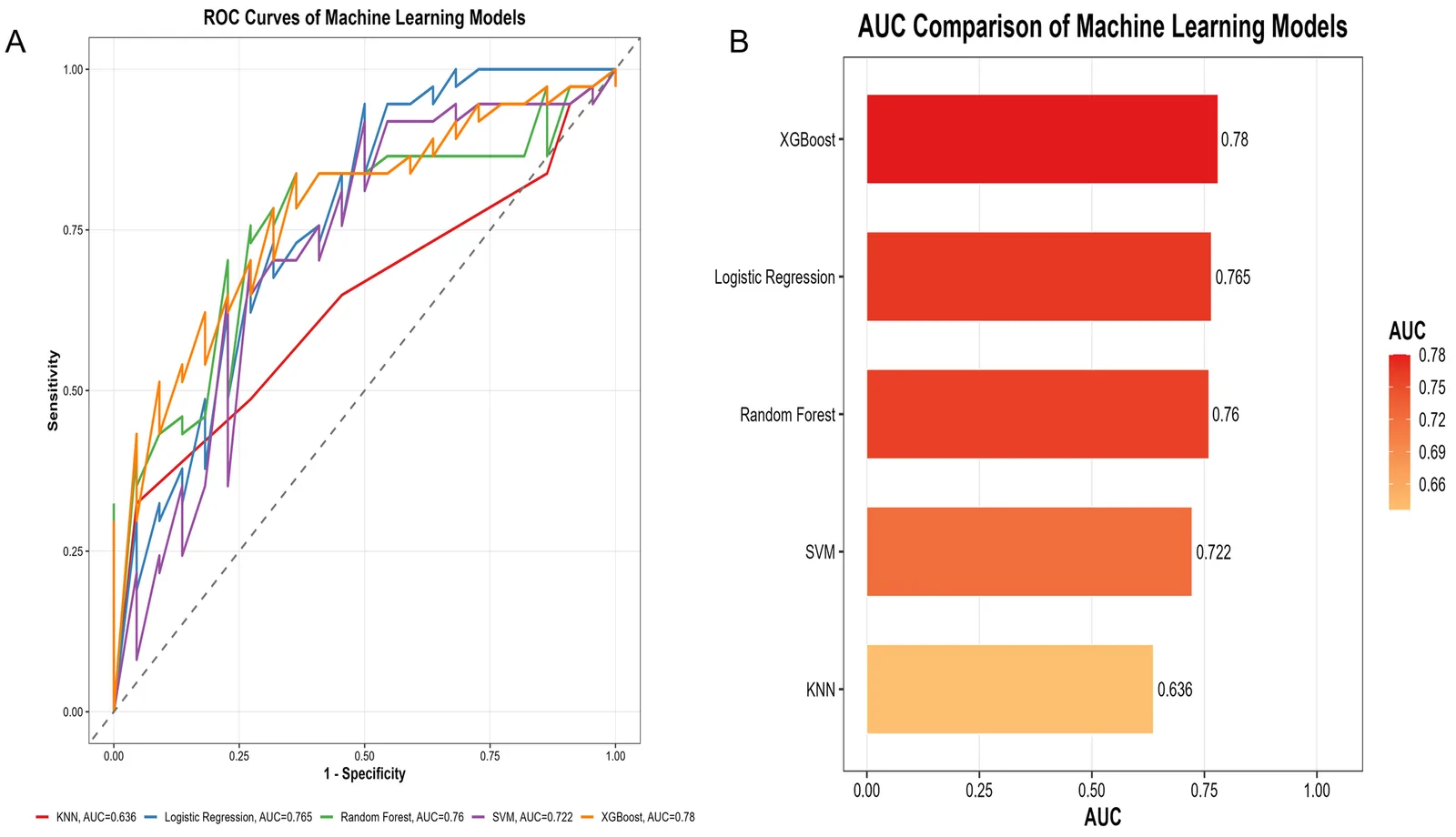
Performance comparison of 5 machine learning model building methods. **(A)** ROC Curves of Machine Learning Models. **(B)** AUc Comparison of Machine Learning Models.

### 3.6 Subgroup analysis of factors associated with swallowing improvement

Exploratory subgroup analyses were performed to examine the associations of planned LFES sessions, time from surgery to LFES initiation, and initial postoperative SSA score with observed swallowing improvement across clinically relevant strata (Table 4). These analyses were conducted as *post hoc* exploratory analyses and were not used for predictor selection, model fitting, or validation. In age-stratified analyses, a greater number of planned LFES sessions was associated with a higher probability of observed swallowing improvement among patients aged <65 years (OR = 1.13, 95% CI: 1.07–1.21; *P* < 0.001), whereas the corresponding association was not statistically significant among those aged ≥65 years (OR = 1.04, 95% CI: 0.95–1.14; *P* = 0.451). After stratification by sex, planned LFES sessions were positively associated with improvement in both female (OR = 1.06, 95% CI: 1.00–1.12; *P* = 0.043) and male patients (OR = 1.12, 95% CI: 1.06–1.20; *P* < 0.001). Among male patients, a longer interval from surgery to LFES initiation showed a borderline inverse association with improvement (OR = 0.84, 95% CI: 0.70–1.00; *P* = 0.054).

**Table 4:** Exploratory subgroup analyses of factors associated with observed swallowing improvement.

Subgroup	Predictor	OR (95% Cl)	P_value
Age			
<65	Planned treatment sessions	1.13 (1.07–1.21)	<0.001
	Time to low-frequency treatment initiation, d	0.94 (0.81–1.09)	0.383
	Initial SSA score after surgery	0.99 (0.95–1.04)	0.801
≥65	Planned treatment sessions	1.04 (0.95–1.14)	0.451
	Time to low-frequency treatment initiation, d	0.94 (0.73–1.21)	0.608
	Initial SSA score after surgery	1.00 (0.95–1.06)	0.937
Sex			
Female	Planned treatment sessions	1.06 (1.00–1.12)	0.043
	Time to low-frequency treatment initiation, d	1.00 (0.86–1.16)	0.961
	Initial SSA score after surgery	1.02 (0.97–1.06)	0.497
Male	Planned treatment sessions	1.12 (1.06–1.20)	<0.001
	Time to low-frequency treatment initiation, d	0.84 (0.70–1.00)	0.054
	Initial SSA score after surgery	0.99 (0.95–1.03)	0.612
Lung infection			
No	Planned treatment sessions	1.12 (1.05–1.19)	<0.001
	Time to low-frequency treatment initiation, d	0.85 (0.72–0.99)	0.048
	Initial SSA score after surgery	1.01 (0.97–1.05)	0.538
Yes	Planned treatment sessions	1.06 (1.01–1.13)	0.032
	Time to low-frequency treatment initiation, d	1.03 (0.88–1.20)	0.730
	Initial SSA score after surgery	0.99 (0.94–1.03)	0.539
Aspiration			
No	Planned treatment sessions	1.09 (1.02–1.16)	0.010
	Time to low-frequency treatment initiation, d	0.93 (0.78–1.09)	0.367
	Initial SSA score after surgery	1.01 (0.97–1.05)	0.710
Yes	Planned treatment sessions	1.09 (1.03–1.15)	0.003
	Time to low-frequency treatment initiation, d	0.93 (0.79–1.09)	0.370
	Initial SSA score after surgery	1.00 (0.96–1.04)	0.920
Combined swallowing training			
No	Planned treatment sessions	1.08 (1.02–1.14)	0.006
	Time to low-frequency treatment initiation, d	0.94 (0.81–1.08)	0.380
	Initial SSA score after surgery	0.99 (0.95–1.03)	0.482
Yes	Planned treatment sessions	1.11 (1.04–1.20)	0.002
	Time to low-frequency treatment initiation, d	0.92 (0.77–1.08)	0.309
	Initial SSA score after surgery	1.03 (0.99–1.08)	0.164

SSA, Standardized Swallowing Assessment.

**Table 5:** Sensitivity analyses of factors associated with observed swallowing improvement.

Sensitivity analysis	Predictor	OR (95%Cl)	P_value
Age <80 years	Age	0.95 (0.93–0.97)	<0.001
	Initial SSA score after surgery	0.99 (0.95–1.02)	0.476
	Aspiration	0.56 (0.28–1.10)	0.092
	Albumin, g/L	1.01 (0.94–1.09)	0.775
	Lung infection	1.15 (0.58–2.32)	0.693
	Time to low-frequency treatment initiation, d	0.87 (0.76–0.99)	0.031
	Planned treatment sessions	1.09 (1.05–1.15)	<0.001
	Combined swallowing training	1.77 (0.88–3.63)	0.113
No lung infection	Age	0.95 (0.92–0.98)	0.001
	Initial SSA score after surgery	1.02 (0.98–1.07)	0.377
	Aspiration	0.50 (0.19–1.29)	0.150
	Albumin, g/L	0.98 (0.88–1.09)	0.695
	Time to low-frequency treatment initiation, d	0.85 (0.70–1.02)	0.096
	Planned treatment sessions	1.13 (1.05–1.23)	0.002
	Combined swallowing training	1.82 (0.61–5.88)	0.294
BMI 18.5–32 kg/m²	Age	0.96 (0.93–0.98)	<0.001
	Initial SSA score after surgery	1.00 (0.96–1.03)	0.818
	Aspiration	0.48 (0.25–0.94)	0.033
	Albumin, g/L	1.01 (0.94–1.09)	0.762
	Lung infection	1.01 (0.51–1.98)	0.982
	Time to low-frequency treatment initiation, d	0.89 (0.79–1.01)	0.067
	Planned treatment sessions	1.10 (1.05–1.15)	<0.001
	Combined swallowing training	1.56 (0.78–3.15)	0.211
Excluding planned LFES sessions	Age	0.96 (0.94–0.98)	<0.001
	Initial SSA score after surgery	0.99 (0.96–1.02)	0.521
	Aspiration	0.53 (0.28–0.99)	0.045
	Albumin, g/L	1.00 (0.94–1.07)	0.997
	Lung infection	0.95 (0.50–1.77)	0.860
	Time to low-frequency treatment initiation, d	0.93 (0.83–1.04)	0.226
	Combined swallowing training	1.31 (0.70–2.45)	0.403

SSA, Standardized Swallowing Assessment.

Similar positive associations for planned LFES sessions were observed among patients without postoperative pulmonary infection (OR = 1.12, 95% CI: 1.05–1.19; *P* < 0.001) and those with pulmonary infection (OR = 1.06, 95% CI: 1.01–1.13; *P* = 0.032), among patients without aspiration (OR = 1.09, 95% CI: 1.02–1.16; *P* = 0.010) and those with aspiration (OR = 1.09, 95% CI: 1.03–1.15; *P* = 0.003), and among those without (OR = 1.08, 95% CI: 1.02–1.14; *P* = 0.006) and with additional structured swallowing training (OR = 1.11, 95% CI: 1.04–1.20; *P* = 0.002). A longer interval to LFES initiation was associated with a lower probability of improvement only among patients without postoperative pulmonary infection (OR = 0.85, 95% CI: 0.72–0.99; *P* = 0.048). Initial postoperative SSA score was not significantly associated with the outcome in any of the examined strata. Overall, the point estimates for planned LFES sessions were generally greater than 1 across the examined subgroups, although statistical significance varied between strata. Given the exploratory nature and limited sample sizes of these analyses, differences in statistical significance should not be interpreted as evidence of effect modification in the absence of formal interaction testing. These findings represent prognostic associations within an LFES-treated cohort and should not be interpreted as evidence that increasing LFES exposure improves swallowing outcomes or that LFES treatment benefit differs between subgroups.

### 3.7 Sensitivity analyses of predictors associated with observed swallowing improvement

To assess the robustness of the principal prognostic associations, *post hoc* sensitivity analyses were conducted under three alternative sample restrictions: exclusion of patients aged ≥80 years, exclusion of patients with postoperative pulmonary infection, and restriction of the cohort to patients with a BMI of 18.5–32 kg/m^2^. These analyses were conducted separately from the prediction-model development and validation workflow and were not used for predictor selection or model tuning. After excluding patients aged ≥80 years, older age was associated with a lower probability of meeting the prespecified swallowing-improvement criteria (OR = 0.95, 95% CI: 0.93–0.97; *P* < 0.001), as was a longer interval from surgery to LFES initiation (OR = 0.87, 95% CI: 0.76–0.99; *P* = 0.031), whereas a greater planned number of LFES sessions was associated with a higher probability of improvement (OR = 1.09, 95% CI: 1.05–1.15; *P* < 0.001). Among patients without postoperative pulmonary infection, older age remained associated with a lower probability of improvement (OR = 0.95, 95% CI: 0.92–0.98; *P* = 0.001), whereas a greater planned number of LFES sessions was associated with a higher probability of improvement (OR = 1.13, 95% CI: 1.05–1.23; *P* = 0.002). The association for time to LFES initiation was directionally negative but did not reach statistical significance (OR = 0.85, 95% CI: 0.70–1.02; *P* = 0.096). When the analysis was restricted to patients with a BMI of 18.5–32 kg/m^2^, older age (OR = 0.96, 95% CI: 0.93–0.98; *P* < 0.001) and aspiration (OR = 0.48, 95% CI: 0.25–0.94; *P* = 0.033) were associated with a lower probability of improvement, whereas a greater planned number of LFES sessions was associated with a higher probability of improvement (OR = 1.10, 95% CI: 1.05–1.15; *P* < 0.001). Time to LFES initiation showed a borderline inverse association (OR = 0.89, 95% CI: 0.79–1.01; *P* = 0.067). Other covariates were not statistically significant in these restricted analyses. Overall, the direction of the associations for age and planned LFES sessions was generally consistent across the restricted analyses, whereas the associations for aspiration and time to LFES initiation were less consistent. Because the planned number of LFES sessions represents a clinician-determined treatment-plan variable, an additional sensitivity model was fitted after excluding this predictor. The validation AUC decreased from 0.767 for the primary eight-predictor model to 0.673 for the seven-predictor model excluding planned LFES sessions, suggesting that the planned-session variable contributed predictive information in this cohort. However, this finding should not be interpreted as evidence that prescribing a greater number of LFES sessions improves swallowing outcomes, because the variable may reflect baseline clinical assessment and treatment-planning decisions rather than a causal treatment effect. These sensitivity analyses therefore support interpretation of the findings as prognostic associations within an LFES-treated cohort rather than estimates of LFES efficacy (Table 5).

### 3.8 Exploratory analysis of individual components of swallowing improvement

To further characterize the composite swallowing-improvement outcome and assess its reproducibility, the independently reconstructible FOIS- and SSA-defined components were examined separately. Among the 200 patients, 84 (42.0%) achieved an increase of ≥2 levels in FOIS and 123 (61.5%) achieved a reduction of ≥9 points in SSA. Eighty-three patients (41.5%) fulfilled both criteria, 40 (20.0%) fulfilled only the SSA criterion, and one (0.5%) fulfilled only the FOIS criterion. Among the 126 patients meeting the composite swallowing-improvement outcome, 84 (66.7%) met the FOIS criterion and 123 (97.6%) met the SSA criterion, indicating that the SSA-defined component accounted for most composite responders. Two composite responders met neither the FOIS nor SSA threshold. Because a separate structured post-rehabilitation aspiration/choking variable was not retained in the analytical dataset, the aspiration/choking component and its overlap with the other components could not be independently reconstructed from the retained analysis file alone. To verify these two cases, Jinfen Hu re-reviewed the original source records, and the original classification of swallowing improvement based on documented improvement in aspiration/choking was confirmed in both patients (Supplementary File).

In exploratory component-specific sensitivity analyses, older age (OR = 0.97, 95% CI: 0.96–0.99; *P* = 0.009), aspiration (OR = 0.53, 95% CI: 0.29–0.97; *P* = 0.040), and a longer interval from surgery to LFES initiation (OR = 0.87, 95% CI: 0.78–0.97; *P* = 0.012) were associated with a lower probability of FOIS-defined improvement. The association between the planned number of LFES sessions and FOIS-defined improvement did not reach statistical significance (OR = 1.04, 95% CI: 1.00–1.08; *P* = 0.076). For SSA-defined improvement, older age was associated with a lower probability of improvement (OR = 0.96, 95% CI: 0.94–0.98; *P* < 0.001), whereas a greater planned number of LFES sessions was associated with a higher probability of meeting the SSA-defined improvement criterion (OR = 1.09, 95% CI: 1.05–1.14; *P* < 0.001). Aspiration showed a directionally negative but statistically nonsignificant association with SSA-defined improvement (OR = 0.55, 95% CI: 0.29–1.05; *P* = 0.070). These component-specific analyses were exploratory sensitivity analyses intended to assess the robustness of the composite outcome and were not used to redefine the primary outcome or to modify the prediction model (Supplementary File). The observed associations should be interpreted as prognostic rather than as evidence of differential LFES treatment effects.

## 4 Discussion

This single-center retrospective study analyzed 200 patients with dysphagia after tracheal reconstruction who received LFES-containing rehabilitation. Univariable and multivariable logistic regression analyses were used to examine prognostic factors associated with observed swallowing improvement, and a clinical prediction model was developed and internally validated. Older age and aspiration were independently associated with a lower probability of meeting the prespecified swallowing-improvement criteria, whereas a greater number of LFES sessions planned before rehabilitation initiation was associated with a higher probability of improvement. The latter should be interpreted as a prognostic association involving a clinician-determined treatment-plan variable rather than as evidence of a dose-response relationship or causal treatment effect. The logistic regression model demonstrated moderate discrimination and generally acceptable calibration, although discrimination decreased after bootstrap correction for optimism. The directions of the principal associations were generally consistent across the sensitivity analyses, while the exploratory subgroup findings should be interpreted cautiously because of limited subgroup sample sizes and the absence of formal evidence of effect modification. XGBoost achieved a numerically slightly higher validation AUC than logistic regression, but the absolute difference was small. Given its interpretability and the exploratory nature of the machine-learning comparisons, logistic regression was retained as the primary prognostic model. Overall, this study identified prognostic factors associated with observed swallowing improvement within an LFES-treated cohort and provides a preliminary framework for prognostic risk stratification and identification of patients who may warrant closer monitoring and reassessment during rehabilitation. External validation is required before the model can be considered for routine clinical use.

Older age was independently associated with a lower probability of meeting the prespecified swallowing-improvement criteria among patients receiving LFES-containing rehabilitation. This finding may reflect the broader effects of aging on swallowing recovery rather than an age-specific difference in the therapeutic effect of LFES. Aging is associated with reductions in swallowing-muscle strength, neuromuscular function, pharyngeal sensation, and swallowing-reflex sensitivity, which may impair the recovery of swallowing function after airway surgery (15–18). Older patients may also have a greater burden of comorbidities, poorer nutritional reserves, frailty, and reduced postoperative recovery capacity, potentially contributing to less favorable swallowing outcomes. These findings suggest that older patients may require closer swallowing-function monitoring, early multidisciplinary assessment, and rehabilitation plans that incorporate structured swallowing training, nutritional support, pulmonary rehabilitation, and aspiration-prevention strategies. However, because the study did not include a comparator group without LFES, it cannot determine whether age modifies the treatment effect of LFES or whether older patients respond less favorably specifically to LFES.

Aspiration was associated with a lower probability of observed swallowing improvement. Impaired laryngeal protection, incomplete glottal closure, reduced pharyngeal sensation, and swallowing discoordination may compromise swallowing safety and increase aspiration risk (19–22). Following tracheal reconstruction, factors such as surgical site traction, airway reconstruction, postoperative edema, and injury from endotracheal intubation or tracheostomy can further impair airway protection (23). In addition, recurrent laryngeal nerve injury or vocal cord dysfunction may cause hoarseness, dysphagia, weak cough, and heightened aspiration risk, consistent with our finding that was associated with a lower probability of observed improvement within this treated cohort (24, 25). Therefore, a comprehensive rehabilitation strategy is recommended for post-tracheal reconstruction patients, including food texture modification, guidance on eating posture, airway protection training, pharyngeal sensory stimulation, and prevention of pulmonary infections.

A higher planned number of LFES sessions was associated with a greater probability of observed swallowing improvement; however, this finding should be interpreted cautiously. Because the number of sessions was determined by the treating clinician before rehabilitation, it may have incorporated information on baseline clinical condition, anticipated rehabilitation potential, expected adherence, or other factors not fully captured by the measured covariates. Thus, this variable may be subject to confounding by indication and is better regarded as a treatment-planning-related prognostic marker rather than a biological determinant of recovery or an estimate of treatment effect. Exploratory component-specific analyses further showed that this association was more evident for SSA-defined improvement, whereas the association with FOIS-defined improvement was not statistically significant. Importantly, excluding planned LFES sessions from the primary multivariable model did not materially alter the associations of age or aspiration with the composite outcome, supporting the stability of these baseline prognostic factors.

Although repetitive neuromuscular stimulation may plausibly require sufficient cumulative exposure to achieve functional adaptation, the present observational data cannot establish a true LFES dose–response relationship. Previous studies of electrical stimulation for dysphagia have reported substantial heterogeneity in stimulation parameters, treatment frequency, session duration, and overall treatment intensity (26), further limiting conclusions regarding an optimal treatment dose. Accordingly, the observed association between planned LFES sessions and swallowing improvement should be considered hypothesis-generating. Prospective studies using standardized treatment protocols and accurately recorded treatment exposure are needed to determine whether treatment intensity or course completion independently contributes to swallowing recovery.

The prediction model developed in this study showed moderate discrimination and generally acceptable calibration in the training and validation sets. Bootstrap internal validation indicated some reduction in discrimination after correction for optimism, underscoring the need for cautious interpretation. Exploratory decision curve analysis suggested potential net benefit across portions of the evaluated threshold-probability range, although this should not be interpreted as evidence of established clinical utility. In the comparative machine-learning analysis, XGBoost achieved a numerically slightly higher validation AUC than logistic regression, whereas the absolute differences among several models were small. Given its interpretability and the exploratory nature of the machine-learning comparison, logistic regression was retained as the primary prognostic model. The potential clinical value of this model lies in its use of routinely available variables to estimate the probability of observed swallowing improvement among patients receiving LFES-containing rehabilitation. Such estimates may help support preliminary prognostic risk stratification and identify patients who may warrant closer monitoring or reassessment during rehabilitation. However, the model was not developed to determine LFES eligibility, prescribe treatment intensity, or estimate individual treatment benefit, and external validation is required before consideration for routine clinical use.

Several limitations should be acknowledged. First, this was a single-center retrospective study and is therefore subject to potential selection bias, information bias, and residual confounding. Second, variation in LFES protocols, planned treatment schedules, adherence, and concurrent swallowing rehabilitation may have contributed to heterogeneity in observed outcomes. In particular, the planned number of LFES sessions was determined by treating clinicians before rehabilitation and may reflect clinical judgment or confounding by indication; its association with improvement should therefore not be interpreted as a causal dose-response effect. Third, although an outcome-stratified training/validation split and bootstrap internal validation were used, no external validation was performed, and the model's transportability to other centers and patient populations remains uncertain. Fourth, although the FOIS- and SSA-defined components of the composite outcome could be independently reconstructed, a separate structured post-rehabilitation aspiration/choking variable was not retained in the analytical dataset. The two composite responders who met neither the FOIS nor SSA threshold were re-reviewed against the original source records and their classifications were confirmed, but the aspiration/choking component could not be independently analyzed in the same manner as the FOIS and SSA components. Fifth, the model relied primarily on routinely collected clinical and swallowing-related variables and did not incorporate more detailed objective assessments such as videofluoroscopic swallowing studies, fiberoptic endoscopic evaluation of swallowing, laryngoscopic findings, surface electromyography, or imaging-derived features. Future multicenter prospective studies incorporating external validation and, where feasible, multimodal swallowing assessments are needed to determine whether the model provides clinically useful incremental information.

## 5 Conclusion

Among patients receiving LFES-containing rehabilitation after tracheal reconstruction, older age and aspiration were associated with a lower probability of meeting the prespecified swallowing-improvement criteria, whereas a greater number of LFES sessions planned before rehabilitation initiation was associated with a higher probability of improvement. The prediction model demonstrated moderate discrimination and may provide a preliminary framework for prognostic risk stratification and identification of patients who may warrant closer monitoring and reassessment during rehabilitation. External validation is required before routine clinical application.

## Data Availability

The raw data supporting the conclusions of this article will be made available by the authors, without undue reservation.
